# T1 mapping in Becker muscular dystrophy patients detects diffuse microfibrosis prior to evidence of late gadolinium enhancement or cardiac dysfunction

**DOI:** 10.1186/1532-429X-18-S1-P261

**Published:** 2016-01-27

**Authors:** Lindsay D Urbinelli, Ryan A Moore, Jacob Mathew, James Starc, Mantosh Rattan, John L Jefferies, Michael D Taylor

**Affiliations:** 1 grid.239573.90000000090258099Pediatric Cardiology, Cincinnati Children's Hospital, Cincinnati, OH USA; 2 grid.239573.90000000090258099Radiology, Cincinnati Children's Hospital, Cincinnati, OH USA

## Background

Becker muscular dystrophy (BMD) is allelic with Duchenne muscular dystrophy and represents a milder skeletal muscle phenotype; however, cardiac dysfunction remains a significant clinical problem. Cardiac involvement in BMD precedes skeletal muscular decline, and cardiomyopathy often leads to death before the age of 60 years. Cardiac magnetic resonance (CMR) is used for ventricular function assessment and myocardial tissue characterization. Late gadolinium enhancement (LGE) in BMD patients is interpreted as a sign of irreversible macrofibrosis, and often prompts changes in cardiac management to try and limit fibrosis progression. T1 mapping is a more sensitive biomarker of diffuse microfibrosis and may offer an opportunity for earlier therapeutic intervention. Currently, myocardial T1 values in BMD patients are unknown. The purpose of this study is to measure T1 values in BMD patients to determine the prevalence of diffuse myocardial microfibrosis, and its relation to LGE and cardiac dysfunction.

## Methods

Twenty-nine BMD patients (mean age 15 ± 5 years; range 8-33 years) underwent CMR for assessment of ventricular function and gadolinium-based tissue characterization. Native myocardial T1 time and post-contrast myocardial T1 ratios (myocardial T1/blood pool T1) were analyzed and compared to published normal values. Additionally, native T1 values and post-contrast myocardial T1 ratios were compared between BMD patients with and without LGE.

## Results

Mean native T1 values were significantly prolonged in BMD patients compared to published normal values (1049 ± 64 msec vs. 953 ± 23 msec; p = <0.001). In addition, the post-contrast T1 ratio (myocardial T1/blood pool T1) was significantly decreased in BMD patients compared to published normal values (1.37 +/- 0.32 vs 1.78 +/- 0.10, p ≤ 0.001). Approximately 31% of BMD patients were positive for LGE, with the basal and mid-ventricular inferolateral segments being the most commonly involved. In BMD patients without LGE, native T1 times remained significantly prolonged and post-contrast T1 ratios remained significantly decreased (p ≤ 0.001 for both). Despite the universally abnormal T1 values, only 14% of BMD patients had evidence of LV dysfunction with an ejection fraction < 55%. Extracellular volumes (ECV) based on T1 mapping results were unable to be calculated due to lack of patient hematocrit data in relation to date of scan.

## Conclusions

T1 mapping in BMD patients demonstrated abnormal pre- and post-contrast T1 values suggesting diffuse myocardial fibrosis and was a more sensitive marker of fibrosis compared to LGE. Detecting diffuse microfibrosis prior to development of scar and cardiac dysfunction could allow for earlier antifibrosis treatment initiation, which might improve cardiovascular morbidity and mortality in the muscular dystrophy population.

